# Health Technology Assessment of Continuous Glucose Monitoring Systems for Paediatric Patients

**DOI:** 10.3390/children12081088

**Published:** 2025-08-19

**Authors:** Martina Andellini, Riccardo Schiaffini, Massimiliano Angelini, Leandro Pecchia, Matteo Ritrovato

**Affiliations:** 1 School of Engineering, University of Warwick, Coventry CV4 7AL, UK; 2 Bambino Gesù Children’s Hospital IRCCS, 00163 Rome, Italy; 3 Endocrinology and Diabetes Unit, Bambino Gesù Children’s Hospital IRCCS, 00163 Rome, Italy; 4 School of Engineering, Università Campus Bio-Medico di Roma, Via Álvaro del Portillo, 21, 00128 Rome, Italy

**Keywords:** health technology assessment, type 1 diabetes, continuous glucose monitoring, multicriteria decision analysis

## Abstract

Objectives: Type1 diabetes (T1D) is one of the most common chronic diseases in pediatric age. Continuous glucose monitoring (CGM) has been shown to improve glycaemic control in adults compared to self-monitoring of blood glucose (SMBG); however, evidence about its use in the pediatric field is limited and fragmented and needs to be improved. This paper aims to address all the critical aspects linked to the use of CGM in a pediatric population while also describing a methodology for conducting health technology assessment (HTA) to support the decision-making process. Methods: The use of CGM and SMBG in a pediatric population was compared by using a decision-making support tool (DoHTA method). Twenty-seven Key Performance Indicators (KPIs) were identified, defining safety, clinical effectiveness, organizational, patient perspective, and economic aspects. Performance scores for both monitoring systems were assessed based on these KPIs, leading to a final comparative ranking. Results: CGM demonstrated a 29.3% performance advantage over SMBG, highlighting its benefits in terms of clinical effectiveness, patient perspectives, safety, and economic evaluation. No substantial differences were identified in terms of organizational aspects. Conclusions: This study critically evaluates the benefits and drawbacks of the use of CGM in a pediatric population. It integrates the assessment of the clinical effectiveness with the organizational aspects, the cost, the patient perspective, and the safety, providing a valuable proof of evidence as well as a reliable and transferable method for conducting decision-making processes in a hospital setting.

## 1. Introduction

Type one Diabetes (T1D) has been one of the most common chronic diseases affecting children and adolescents over the past two decades. The European Childhood Diabetes Registry shows an increment in the incidence rate of 3.4% per annum in Europe, forecasting that it will double over the next 20 years [1]. Furthermore, recent studies indicate that over the past two decades, the onset of diabetes has increasingly occurred at younger ages [2].

The steady increase in the incidence of T1D within children and adolescents is associated with lifelong increases in morbidity and mortality, leading to significant implications in healthcare planning and delivery. Pediatrics patients with T1D are at greater risk of developing acute complications, compared to adult patients [3].

A key aspect is achieving better blood glucose control, which has been shown to reduce short- and long-term complications through the use of advanced insulin delivery systems and sensor technologies. Therefore, optimal management, including reducing HbA1c levels and maintaining stable glycaemic control, is crucial for pediatric patients [4]. Several studies confirm that effective management of diabetes during the pediatric age not only reduces the risk of adverse events but is able to delay the onset of long-term complications [5,6].

Managing T1D requires daily SMBG. However, pediatric and adolescent patients often struggle with SMBG and self T1D management not only due to dietary changes but also because SMBG requires invasive and cumbersome finger-prick tests. This can negatively affect compliance with glucose monitoring [7].

As an alternative, CGM offers real-time glucose measurements based on interstitial fluid glucose levels, helping reduce the burden of self-care by minimizing the need for frequent finger pricks.

The use of CGM devices has been associated with significant improvements in glucose control among diabetic patients [8,9,10,11,12,13]. The major scientific societies (ISPAD, ADA and EASD) support the use of CGM as the standard of care for glucose monitoring in type 1 diabetes [14,15,16]. Its use in children and adolescents with T1D has the potential to revolutionize diabetes management [17] by improving the quality of life [18,19,20].

However, while robust evidence supports the effectiveness of CGM in adults with type 1 and type 2 diabetes, RCTs in children are limited and fragmented [21]. The available evidence in pediatric population is of moderate quality, relying primarily on cohort or registry studies and expert consensus or clinical experience. In a large randomized controlled trial of 322 patients (adults and children), the use of CGM was associated with improved glycaemic control in adults but not in children and adolescents [22,23,24]. Another randomized control trial concluded that among adolescents and young adults with T1D, the use of CGM instead of SMBG resulted in a small but statistically significant improvement in glycaemic control over 26 weeks [25,26].

Among ten observational studies investigating HbA1c variation associated with the use of CGM [12,22,23,27,28,29,30,31,32], four showed improvements in glycaemic control [23,28,32,33], five showed improvement in glycaemic control among consistent CGM users [12,22,27,30,32], and one found no statistically significant changes in HbA1c between groups [31]. Two meta-analyses [34,35] showed a reduction in hypoglycaemic episodes in the CGM group compared to the SMBG group. Accordingly, a real-world study which tracked pediatric and adult patients who switched between CGM and SMBG highlighted that switching from SMBG to CGM was associated with a statistically significant drop in median HbA1c in children/adolescents [36]. In addition, four non-randomized control trials evaluated the use of CGM in pediatric patients with T1D; three of them showed no significant differences in glycaemic parameters between CGM and SMBG groups [12,37,38], while one study reported that CGM was effective in improving glycaemic control in youth [39].

Another important aspect that should be assessed is the patient perspective. The use of CGM enables the detection of asymptomatic hypoglycaemia and hyperglycaemia, improving the quality of life of patients with type 1 diabetes, reducing worry about fluctuating blood sugars, enhancing the sense of safety with children who may not recognize or communicate symptoms of low and/or high blood glucose, and minimizing pain associated with frequent finger-pricks.

However, to ensure these benefits, children newly diagnosed with T1D, along with their families, require comprehensive diabetes education from an interprofessional pediatric diabetes healthcare team including a pediatric endocrinologist, dietitian, diabetes nurse educator, and mental health professional. The primary goal is to train and empower families to effectively manage the T1D condition [20]. This includes insulin administration and dosage adjustment, blood glucose and ketone monitoring, sick-day management to prevent diabetic ketoacidosis (DKA), nutritional therapy, physical activity planning, and strategies for recognizing and treating hypoglycaemia. Given the complex physical, developmental, and emotional needs of children, specialized care is crucial to achieving optimal long-term outcomes.

The objective of this study is to conduct a comprehensive Health Technology Assessment study by comparing the management of type 1 diabetes in children under 18 years old who use CGM versus SMBG. The aim is to assess different aspects of CGM use in children, integrating the clinical effectiveness of device use with the organizational impact, the patients’ quality of life, the cost-effectiveness, and the patient perspective.

The paper describes in detail the evaluation process that has been conducted within our hospital context, offering a clear examination of the organizational impact and the clinical benefits for patients, and the patient perspective.

Thanks to the HTA process, which represents a multidisciplinary process and a method of evidence synthesis, it was possible to measure the performance of glucose monitoring systems, focusing not only on the clinical outcomes but also complementing the existing evidence with organizational dimensions and patient perspectives, including quality of life and cost-effectiveness. This multidimensional evaluation provides a broader understanding of the overall impact of technology on the healthcare system and its stakeholders.

## 2. Materials and Methods

### 2.1. Setting and Inclusion Criteria

Thirty-six pediatric patients who were diagnosed with T1D were recruited at the Endocrinology Department of Bambino Gesù Children’s Hospital. Both male and female subjects diagnosed with T1D, aged less than 18 years old, who are currently under the care of the Unit of Endocrinology and Diabetes of Bambino Gesù Children’s Hospital, Rome, Italy, and who use CGM systems or SMBG were eligible to be involved in the study. Patients with coexistence of celiac disease or non-diabetic hypoglycaemia or cardiovascular pathologies and cardiac arrhythmias were excluded. Furthermore, patients who were pregnant or became pregnant during the study were also excluded. Out of the 36 patients, 22 used CGM sensors (15 patients used FreeStyle Libre (Abbott, Alameda, CA, USA) and 7 patients used and Dexcom (Dexcom, San Diego, CA, USA) sensors), whereas 14 used SMBG. The mean age of the CGM group was 12.4 ± 3.6 years and the mean age of the SMBG group was 13 ± 3.3 years.

### 2.2. Health Technology Assessment Process

The Health Technology Assessment process was conducted by using a decision-making support tool (DoHTA method) that integrates the MCDA by using the Analytic Hierarchy Process (AHP) and the EUnetHTA CoreModel© [40].

This method proposes to organize the collected evidence in different KPIs made up of the domains, which are arranged in a hierarchical decision structure. The idea is that a decision problem can be decomposed into a hierarchy of more easily solved sub-problems, each of which can be measured and analyzed independently. The AHP mathematical process, using pairwise comparisons, the eigenvector method for deriving weights, and a method to verify the “consistency” of judgments, is able to integrate all KPI evaluations in a final numerical result, which represents the alternatives’ relative ability to achieve the decision goal. It is characterized by eight steps. The whole process is described in Supplementary S1.

To assess the technologies under evaluation, a multidisciplinary working group was involved in the study. The working group created was made up of experts who are potentially impacted by the use of technologies: three medical doctors (endocrinologists), one psychologist, and two biomedical engineers.

#### 2.2.1. Definition of the Problem and Identification of Technologies

The objective of this first step is to define the decision problem and to identify the technologies to be compared.

#### 2.2.2. Evidence Gathering

After defining the decision problem, a general literature search was preliminarily carried out, aimed at gathering evidence on the two technologies, obtaining a general overview of the subject, and defining the main criteria representing discriminant assessment factors between the technologies under comparison and the state of the art. The search focused on papers regarding safety, efficacy, costs, organizational, and technical features of the two technologies under comparison. Evidence was also gathered from grey literature.

#### 2.2.3. Hierarchy Construction

In this phase, the evaluation criteria were identified to assess the health technologies. The idea was that a decision problem can be decomposed into a hierarchy of more sub-problems, each of which can be measured and analyzed independently.

The main evaluation criteria (Domains) were identified, by the working group, among those singled out by the EUnetHTA Core Model© [41,42], which represents the European guideline for each HTA process. Following the EUnetHTA Core Model, each technological innovation could be analyzed according to nine domains: the current use of the technology, safety, clinical effectiveness, costs, social, ethical, legal, technical, and organizational aspects.

Each domain of the EunetHTA Core Model is described by different topics that represent more specific aspects within the domain represented.

More specifically, the “goal of the decision” was split into the main evaluation criteria (i.e., the domains), with each one divided in turn into KPIs, covering all the pertinent aspects to be analyzed in the assessment of the different glucose monitoring systems. According to AHP, the KPIs identified were arranged in a hierarchy decision tree.

#### 2.2.4. Alternatives Performances Evaluation

With the hierarchical decision tree completed, the performances of the two technologies were measured with respect to each KPI. The performances of the two technologies were measured with different methods depending on the number of observations and whether the KPI is measurable or not.

Data were collected from hospital registries, Electronic Health Records (EHR), from CGM, and through patients and medical doctors’ interviews. The data collection method per each KPI identified is shown in Supplementary S2.

The non-parametric Mann–Whitney U test (significance level *p* < 0.05) was used to compare the performance of alternative technologies with respect to the measurable KPIs (see Supplementary S2). All statistical analyses were performed using SPSS Statistics software version 29.

Moreover, according to the AHP method, professionals were asked to translate into the Saaty scale [43] (through a series of pairwise comparisons) the performance values of the two glucose monitoring systems measured through statistical methods, providing the clinical significance of the statistical analysis results. In this way, the statistical analysis results were interpreted considering the population and the hospital setting in question.

The remaining KPIs, which are not directly measurable, were directly assessed through pairwise comparison according to AHP method. Each component of the working group expressed their own judgement, comparing the performances of the two glucose monitoring systems, in order to establish which of the alternative technologies has the higher performance and at which level with respect to the KPIs.

All the qualitative answers to the pairwise comparisons were then translated into numbers, according to the AHP method, through the Saaty scale [44].

#### 2.2.5. Weighting of Criteria

The weights for each criterion were then computed. The aim of this step is to define the weight of each indicator within the evaluation. The mathematical treatment of this step matches the AHP method. During this phase, each member of the working group has to answer to a set of KPIs’ pairwise comparisons.

#### 2.2.6. Integration of Results

Following the AHP method, the absolute performance values of each KPI were then weighted by the relative domain’s weight in order to obtain the final alternative technologies scores.

#### 2.2.7. Sensitivity Analysis

Sensitivity analysis was then performed to test the stability and the robustness of the alternatives’ ranking. This analysis enables the identification of the sources of uncertainty and the evaluation of their impact on the robustness of the assessment results. Sensitivity analysis was carried out by calculating the minimum changes in values of each criterion needed to reverse the current ranking of alternative technologies [45].

## 3. Results

### 3.1. Definition of the Problem and Identification of Technologies

The objective of this work is to compare the management of T1D in children younger than 18 years by comparing the use of CGM and standard glucometer by evaluating the hospital workflow and the organizational aspects, the effectiveness of devices uses, the quality of life, the cost-effectiveness, and the social perspective.

### 3.2. Evidence Gathering and Hierarchy Construction

The working group decided to include in the analysis the five domains from the EunetHTA Core Model that are able to highlight the difference and that represent discriminant factors among the technologies under evaluation: safety, clinical aspects, costs and economic evaluation, organizational impact, and patient perspective.

Through the information gathered from the general literature search, integrated with the information from health professionals, the working group defined 27 KPIs, which represent the main factors for discriminating against the technologies under evaluation.

**Figure 1:**
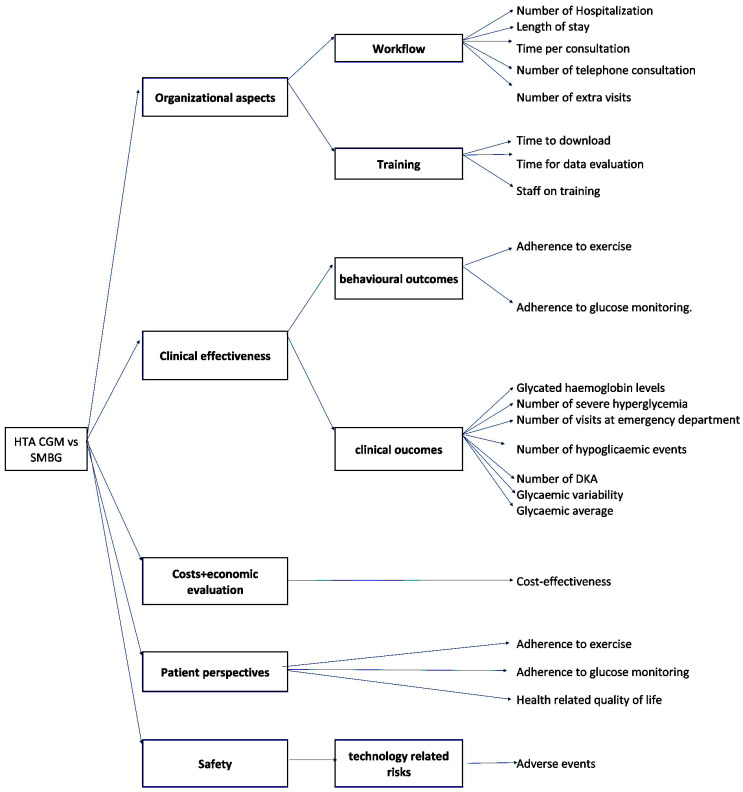
Decision hierarchy tree.

Each KPI was then arranged in the hierarchical decision tree (Figure 1) as Lev-1 and Lev-2 KPIs, aiming at better describing each domain in detail for the specific analysis.

More specifically, the organizational aspects were described by the Lev-1 KPIs: “Workflow” and “Training”. The “Workflow” was evaluated by collecting data on the hospitalization rate, the length of stay, the time per consultation, the number of telephone consultations, and the number of extra visits. The “Training” was evaluated by assessing the time to download data, time for data evaluation, and the training of the staff.

To evaluate the Clinical effectiveness, two main aspects were considered: the “Behaviour outcomes” and the “Clinical Outcomes”. The former were assessed by measuring the adherence to exercise and the adherence to glucose monitoring, whereas the latter was evaluated by collecting data on Hba1c level, number of visits to the emergency department, number of severe hyperglycaemia events, number of hypoglycaemic events, number of DKA, glycaemic variability, and glycaemic average.

Patient perspective was also included in the analysis by assessing the Quality of Life, adherence to therapy, and adherence to exercise.

Regarding the Cost and Economic evaluation, a cost-effectiveness analysis was performed by evaluating the costs and health benefits of using CGM devices instead of SMBG over a 16-year timeline.

Finally, safety was evaluated through the analysis of the technology-related risk, especially in the ability to capture hypoglycaemia and hyperglycaemia events.

### 3.3. Alternatives Performances Evaluation

CGM and SMBG performances were compared using Level-2 KPIs, assessed by professionals via the AHP method through pairwise comparisons based on their professional experience, evidence, and data analysis. The AHP method converted qualitative judgments into numerical scores using the Saaty scale [43,44,46].

Moreover, as professionals’ responses were not only based on their experience but also on data collected and analyzed (also via statistical analysis), this method allowed for an appropriate interpretation of the statistical results, providing the clinical significance of results, and reflecting the impact that the two health technologies have on the specific clinical case for the specific study population and in the specific clinical setting.

Data were collected from EHRs (e.g., hospitalizations, DKA), interviews (e.g., consultation time, adherence to therapy and exercise), and questionnaires (EuroQol 3D3L young for Quality of Life). Clinical metrics like HbA1c, glycaemic variability, and severe event rates were obtained from CGM devices or hospital visits for the SMBG group (further details in Supplementary S2).

Statistical analysis results (Mann–Whitney U test) show that patients who use CGM, compared to patients with SMBG, obtained a statistically significant reduction in glycaemic variability, glycaemic average, and HbA1c. Non-statistically significant reductions in the severe hypoglycaemia and hyperglycaemia events rate were observed for those patients who used CGM, while a statistically significant enhancement of health-related quality of life occurred, resulting in statistical improvement in the health condition due to the use of CGM (Table 1).

Final performance scores were derived using AHP methods by converting the professionals’ judgements into percentage values. The results are presented in Table 2.

More specifically, the organizational aspects analyzed the impact of the technologies’ introduction on the existing hospital workflows together with the training needed. Overall, the use of CGM or SMBG does not seem to have a significant impact on the organizational aspects. However, an important reduction in the hospitalization rate is reported in those patients who use CGM, together with a reduction in the length of stay.

The time per consultation is comparable between patients using SMBG and those using CGM, with a slight increase observed in CGM users, attributable to the additional time required by the medical staff to analyze the greater amount of data generated by the sensor.

The wealth of glucose information provided by the CGM sensor has led to an improvement in glycaemic control related to the incorporation of glucose trends and alerts and the ability for automated insulin delivery, which reduced the number of telephone consultations (10% less than SMBG group) and the number of extra visits.

Regarding the training aspects, the use of CGM has been shown to reduce the time for data download, as it is able to generate a detailed report over a specific range of time within seconds. However, it increases the time for data evaluation and interpretation, given the significantly greater amount of data and information to be analyzed in collaboration with patients.

In accordance with the previous two KPIs, the time spent by members of staff on training in order to learn how to efficiently use CGM systems and efficiently use the online platform to download and generate data reports is higher compared to SMBG, which does not require any further training.

Regarding clinical effectiveness, CGM performs better considering both the behavioural and the clinical outcomes. More specifically, medical doctors registered an increase in the adherence to exercise and adherence to glucose monitoring in those patients who used CGM compared with the SMBG group.

The results also show a statistically significant reduction in glycated hemoglobin levels, glycaemic variability, and glycaemic average in patients with CGM.

Also, the number of severe hyperglycaemic events, the number of hypoglycaemic events and DKA, and consequently the number of visits to the emergency department were reduced in the group of patients who use CGM.

The patient perspective was analyzed by assessing the adherence to therapy, adherence to glucose monitoring, and the health-related quality of life. The results show that the use of CGM enhances patient compliance with therapy and glucose monitoring. The most important KPI from the patients’ perspective is the health-related quality of life for children living with T1D and their families.

The results show that the use of CGM statistically significantly improves multiple measures of children’s quality of life, reducing family stress and improving the sense of control parents related to their child’s condition.

Regarding the cost and economic evaluation, the results showed that the use of CGM in the pediatric population, compared with SMBG, was associated with substantial health benefits. Over the 16-year timeline, CGM use resulted in an average gain of 3.14 quality-adjusted life years (QALYs) per patient at an incremental cost of EUR 45,914.94, yielding an ICER of EUR 14,103.63 per QALY gained, resulting in cost-effectiveness at the Willingness to Pay threshold of 20,000 GBP/QALY established by the National Institute for Health and Care Excellence.

Regarding the safety aspects, CGM seems to be safer than SMBG, particularly in its enhanced ability to detect a greater number of hypoglycaemic and hyperglycaemic events, especially during the night, thereby potentially reducing the incidence of related adverse events.

### 3.4. Weighting of Criteria

**Figure 2:**
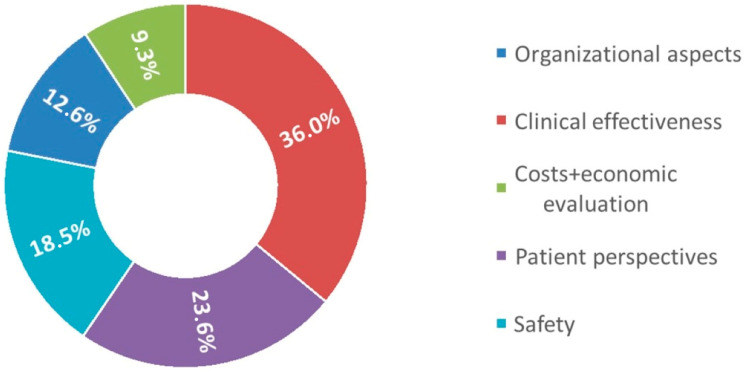
Weighting of criteria. Ring plot illustrating the unified weights’ system (which represents the percentage level of importance of the various domains with respect to the overall evaluation) pertaining to the “domains” layer of the decision tree, as gathered from pairwise comparisons and mathematical calculations in accordance with the AHP method).

The ring plot illustrated in Figure 2 represents the unified weighting system for the domains layer of the decision tree, based on the collective judgements of all the professionals involved, as calculated using the AHP method. More specifically, the ring plot represents the aggregated weights of KPIs assigned by each professional interviewed.

The results showed that the most important aspects to be considered when comparing CGM and SMBG are clinical effectiveness and patient perspectives reaching, respectively, 36% and 23.6% of the total weight, followed by safety (18.5%), the organizational aspects (12.6%), and the cost and economic evaluation (9.3%). Detailed information about each indicator’s weight is listed in Table 2. Detailed information about the calculation of these percentage values is shown in AHP-related literature [37,39,40].

More specifically, within the clinical effectiveness domain, the weight of the “clinical outcomes” is approximately twice that of the “behavioural outcomes”. The clinical outcomes domain includes all the relevant clinical parameters that more or less equally affect the clinical outcomes, such as the glycated hemoglobin levels, number of severe hyperglycaemia number of visits at emergency department, number of hypoglycaemic events, number of DKA, glycaemic variability, and glycaemic average. Within the “behavioural outcomes”, the “adherence to glucose monitoring” is the most representative Lev 2-KPI, which more than doubles the weights of the “adherence to exercise”.

The most important aspect of the patient perspective domain resulted in the health-related quality of life.

Regarding safety, the working group believes that adverse events should be carefully considered. CGM is able to reduce the technological risks, providing programmable alarms to detect hypo- or hyperglycaemic events, increasing the sense of safety, especially for pediatric patients who cannot recognize the symptoms.

Regarding the organizational aspects, the impact that the different monitoring devices have on the hospital processes (e.g., number of hospitalizations, length of stay, time per consultation, number of telephone consultations, and the number of extra visits) weighed slightly more than the training-related aspects, which consider the time to download data, time for data evaluation, and the training of the staff.

Finally, the results of the economic evaluation aimed to compare not only the device costs but the cost-effectiveness analysis, considering the lifetime effects in terms of cost and clinical outcomes over 16 years of follow-up.

### 3.5. Integration of Results

The absolute performance values of each Level-2 KPI were then weighted by the relative domains weight as calculated in the previous step, in order to obtain the alternative technologies’ scores and the final global rank of the two glucose monitoring methods.

**Figure 3:**
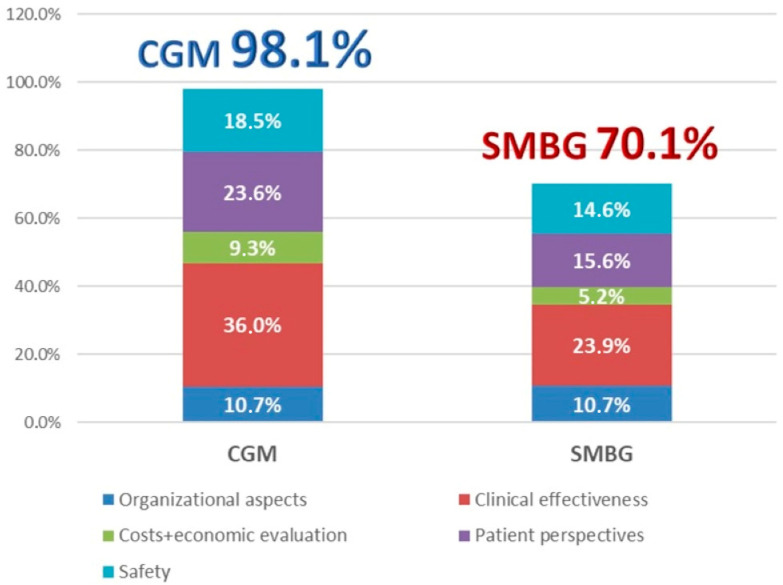
Histogram chart specifying the computed performance (global and per domain) of CGM vs. SMBG.

The final results (absolute performances weighted by the relative domain weight) are shown in the histogram chart (Figure 3) and in Table 2. According to the AHP method [24,25], performances were expressed as percentages and derived from the elaboration of the pairwise comparisons made by all the professionals involved.

As shown in Figure 3, which specifies the computed performance (global and per domain) of the management of T1D via CGM and SMBG, the management of pediatric patients with CGM seems to be the best option compared with SMBG. The final performance value of CGM was 29.3% over that of SMBG. Table 2 (2nd, and 3rd columns) gives detailed information about each indicator’s performance value for the technologies compared and about the relative weight of each element with respect to the overall evaluation (1st column).

### 3.6. Sensitivity Analysis

By design, sensitivity analysis is performed when the final performance scores of different models are close to each other, as a small variation in one parameter can potentially reverse the final outcome. In our case, CGM outperformed SMBG by 29.3%, indicating that the results are robust and reliable.

## 4. Discussion

This work provides a general overview of the most relevant features (identified KPIs) to consider when assessing the use of CGM compared to SMBG in a pediatric population affected by T1D.

The central objective of this study is to synthesize and integrate various dimensions of technology evaluation, including organizational factors, clinical effectiveness, patient quality of life, cost-effectiveness, and social impact, into a unified numerical result.

It discusses the main characteristics of these technologies, as well as the potential implications of their routine use in clinical practice. Additionally, it provides detailed information on the methodology employed to carry out the assessment.

The added value of this study lies in its primary data collection, which captures critical dimensions often underrepresented in pediatric literature, such as quality of life, organizational factors, and patient perspectives, while also confirming the clinical benefits of CGM.

The results shows that the clinical effectiveness and patient perspective are the most important domains within the assessment, together accounting for about 60% of the total evaluation weight.

The clinical effectiveness evaluation provided information on clinical and behavioural outcomes. As already extensively discussed in the introduction, CGM use has been shown to improve glycaemic control in adolescents and young adults, while only a few studies are available for the pediatric population. Our study supports the idea that CGM use significantly improves glycaemic outcomes in terms of glycated hemoglobin levels, glycaemic mean, and glycaemic variability in pediatric patients, also confirming the results of Champakanath et al. [47], which demonstrated that initiating CGM use within the first year of T1D diagnosis provides long-term benefits compared to patients who started later or did not use CGM at all.

Behavioural outcomes are a pivotal aspect of clinical effectiveness, as they have direct implications for health outcomes. This study demonstrated that the use of CGM improves adherence to glucose monitoring and exercise, enhancing glycaemic control and reducing the risk of short- and long-term diabetes-related complications [26].

The patient perspective also plays a crucial role in the assessment of CGM, including adherence to glucose monitoring, exercise, and the health-related quality of life of children with T1D and, consequently, their families.

As no data on the quality of life of children with T1D were retrieved from the literature search, data were collected by administering quality of life questionnaires to the recruited children. The results show that CGM use is associated with a statistically significant increase in QALYs. The major benefits reported by children and their families included avoiding frequent (and painful) fingerstick checks, reducing the fear of hypoglycaemia with predictive alarms, especially in children unable to recognize hypo- or hyperglycaemic symptoms, and improving parental control over their child’s condition [48,49]. Moreover, by preventing major symptomatic hypo- and hyperglycaemic events, CGM use has the potential to improve school attendance, adherence to exercise, and participation in age-appropriate activities [50]. However, despite the health benefits, a proportion of children preferred to continue using SMBG due to distress or embarrassment associated with wearing a technical device and due to local allergic reactions to adhesives.

Regarding costs and economic evaluation, our study confirmed that despite the high cost of CGM devices, improved glycaemic control reduces the risk of short-term acute diabetes-related complications, thus highlighting the technology’s cost-effectiveness.

Organizational aspects also play a critical role in the evaluation. The benefits of CGM use can be measured through several organizational KPIs that assess its impact on hospital workflows. CGM use led to a reduction in the number of telephone consultations and extra visits, although it resulted in longer hospital visit times due to the greater amount of data to analyze compared to SMBG.

The clinical benefits of CGM were also reflected in a reduced hospitalization rate and shorter length of hospital stays, serving as evidence of fewer T1D-related complications requiring hospitalization.

In contrast, SMBG had a positive impact on staff training, as no additional training was required, and it reduced the time needed to download and evaluate patient data during hospital visits, given the smaller volume of collected data.

Finally, safety aspects were also considered in the assessment, concluding that CGM appears to be safer than SMBG, particularly in its ability to detect a significantly higher number of hypoglycaemic and hyperglycaemic events, especially during the night, thereby reducing related adverse events.

## 5. Conclusions

This study presents a comprehensive and multidimensional HTA study of CGM in the real-world pediatric hospital setting. By incorporating primary data collection on organizational and psychosocial dimensions, areas often overlooked in traditional clinical studies, it offers a more holistic evaluation framework. The transparent application of MCDA and AHP enables a structured, replicable model for hospital-based technology adoption, integrating both scientific evidence and the informed judgement of clinical professionals.

The assessment captures not only clinical effectiveness but also patient-reported outcomes, quality of life, organizational implications, cost, and safety. It provides a detailed overview of KPIs within clearly defined domains, thereby supporting informed and balanced decision making.

Furthermore, the methodological framework and its outputs are readily transferable to similar healthcare settings. With appropriate adaptation, they may serve as valuable tools for the evaluation of CGM or comparable technologies in other institutional contexts.

Despite these strengths, some limitations must be acknowledged. The sample size (n = 36) is relatively small, which may affect the generalizability of the findings. Larger, multicentre studies are needed to enhance external validity. The reliance on expert judgement within the AHP model introduces potential bias; including a higher number and a more diverse range of stakeholders could improve the robustness of future assessments. Finally, the use of data from a single hospital constrains the depth of analysis and suggests that future studies may benefit from integrating data from several pediatric hospitals.

In conclusion, this study demonstrates the feasibility and value of a structured, multidimensional HTA approach to evaluate pediatric technologies in real-world hospital settings. It lays the foundation for more robust, inclusive, and transferable models of assessment that can support evidence-informed decision making across healthcare systems.

## Figures and Tables

**Table 1:** Statistical analysis results.

Clinical Effectiveness	CGM (Mean ± Sd)	SMBG (Mean ± Sd)	*p* Value
Glycaemic variability	56.75 (±10.1)	79.3 (±21.1)	0.003 *
HbA1c (mmol/mol)	53.5 (±12.2)	70.2 (±14.2)	0.004 *
Glycaemic average	153.4 (±17.1)	186.4 (±43.6)	0.02 *
Severe hypoglycaemic events rate	0.22% (±0.3%)	0.3% (±0.5%)	0.687
Severe hyperglycaemic events rates	13% (±12%)	18% (±15%)	0.408
Health-related quality of life	91.4 (±4.4)	76.3 (±4.9)	0.09 *

* Statistically significant at *p* < 0.05.

**Table 2:** List of KPIs, with the relative global weights and performances’ percentage scores. Domains are listed in capital bold, Lev-1 KPIs in bold and Lev-2 KPIs in plain text.

		Weight System	Performance CGM	Performance SBGM
**ORGANIZATIONAL ASPECTS**		**12.6%**	**10.7%**	**10.7%**
**Workflow**		**6.6%**	**6.4%**	**5.2%**
	Hospitalization rate	2.2%	2.2%	1.2%
	Length of stay	0.8%	0.8%	0.6%
	Time per consultation	1.0%	0.9%	1.0%
	Number of telephone consultation	1.3%	1.2%	1.3%
	Number of extra visits	1.3%	1.3%	1.0%
**Training**		**6.0%**	**4.3%**	**5.5%**
	Time to download	1.1%	1.1%	0.6%
	Time for data evaluation	3.6%	2.4%	3.6%
	Staff on training	1.3%	0.9%	1.3%
**CLINICAL EFFECTIVENESS**		**36.0%**	**36.0%**	**23.9%**
**Behavioural outcomes**		**12.9%**	**12.9%**	**10.1%**
	Adherence to exercise	3.8%	3.8%	2.9%
	Adherence to glucose monitoring.	9.1%	9.1%	7.2%
**Clinical outcomes**		**23.1%**	**23.1%**	**13.8%**
	Glycated hemoglobin levels	2.1%	2.1%	0.7%
	Number of severe hyperglycaemias	2.2%	2.2%	1.5%
	Number of visits at emergency department	3.1%	3.1%	1.0%
	Number of hypoglycaemic events	5.4%	5.4%	4.8%
	Number of DKA	3.6%	3.6%	2.0%
	Glycaemic variability	3.7%	3.7%	2.1%
	Glycaemic average	3.0%	3.0%	1.7%
**COSTS AND ECONOMIC EVALUATION**		**9.3%**	**9.3%**	**5.2%**
**CEE**		**9.3%**	**9.3%**	**5.2%**
	Cost-effectiveness	9.3%	9.3%	5.2%
**PATIENT PERSPECTIVES**		**23.6%**	**23.6%**	**15.6%**
**Patients**		**23.6%**	**23.6%**	**15.6%**
	Adherence to exercise	4.0%	4.0%	3.1%
	Adherence to glucose monitoring.	6.6%	6.6%	5.2%
	Health related quality of life	13.0%	13.0%	7.3%
**SAFETY**		**18.5%**	**18.5%**	**14.6%**
**Technology related risks**		**18.5%**	**18.5%**	**14.6%**
	Adverse events	18.5%	18.5%	14.6%
**TOTAL**		**100.0%**	**98.1%**	**70.1%**

## Data Availability

Data will be made available upon reasonable request.

## Supplementary Materials

The following supporting information can be downloaded at https://www.mdpi.com/article/10.3390/children12081088/s1, Supplementary S1: HTA process; Supplementary S2: Data collection methods.

## Abbreviations

The following abbreviations are used in this manuscript:

CGM	Continuous Glucose Monitoring
SMBG	Self-monitoring of blood glucose.
HTA	Health Technology Assessment
QALYs	Quality-Adjusted Life Years
KPIs	Key Performance Indicators
MCDA	Multicriteria Decision Analysis
AHP	Analytic Hierarchy Process
EHR	Electronic Health Records
DKA	Diabetic Ketoacidosis
