# Supplementary material for: Health Technology Assessment of Continuous Glucose Monitoring Systems for Paediatric Patients
Source: Children (Basel). 2025 Aug 19;12(8):1088. doi: 10.3390/children12081088 (PMC12384686; doi:10.3390/children12081088)
Supplement: Supplementary file 1 [file children-12-01088-s001.zip › children-3776833-supplementary.pdf]

Supplementary S1: HTA process.

The evaluation of technologies is carried out by using the doHTA method, a decision-making support tool that integrates the EUnetHTA Core Model© (defining the schema for the evaluation of healthcare technology) with the mathematical method of the Analytic Hierarchy Process (AHP). It is possible to primarily structure the methodology into eight phases as shown in Table S1. These phases enable a structured and systematic approach to the evaluation of healthcare technologies, ensuring a decision-making process based on evidence and criteria defined clearly and transparently.

Table S1 Explains in detail the eight phases of a complete HTA process. The first four steps represent the assessment phase, whereas the remaining steps represent the appraisal phase.

**1**	**Definition of the problem and identification of technologies**: In this phase, the decision problem is clearly defined, and the healthcare technologies subject to evaluation are identified.	**ASSESSMENT PHASE**
**2**	**Evidence Gathering**: The collection of evidence generally focuses on aspects related to safety, clinical effectiveness, technical characteristics, organizational, ethical, social, and legal aspects.	
**3**	**Hierarchy Construction.** The Evaluation criteria are identified for assessing healthcare technologies. The problem is broken down into more specific and measurable sub-objectives, Key Performance Indicators (KPIs), to provide a hierarchical structure to the evaluation.	
**4**	**Alternatives performances evaluation**: Alternatives technologies are evaluated against the KPIs defined in the previous phase using empirical data and expert knowledge.	
**5**	**Weighting of criteria:** Criteria are weighted based on their relative importance in the decision problem context using the Analytic Hierarchy Process (AHP).	**APPRISAL PHASE**
**6**	**Integration of results:** The absolute performances are weighted by the relative weight of the specific element to obtain a final numerical result.	
**7**	**Sensitivity Analysis**: to test the robustness and to assess the stability of the results concerning the evaluation assumptions	
**8**	**Decision-making and communication of results**: In this phase, final decisions are made based on the evaluation results and communicated clearly and transparently to all stakeholders.	**RESULTS PRESENTATION**

Supplementary S2: Data collection methods.

Table S2 shows the data collection method per each KPI identified.

Data were collected in different ways depending on the nature of the single parameter.

Data related to the organizational aspects were collected from hospital's database and from interviews with medical staff in order to capture the real organizational issue of the specific setting

More specifically, data on the hospitalization rate, length of stay, number of extra visits, number of visits to the emergency department and number of DKA were collected from EHR of patients recruited (Data collected for each patient was averaged over the years from the onset of T1D for SMBG group and over the years of using CGM device for CGM group.

The time per consultation, the number of telephone consultations, time to download data, time for data evaluation and the training of the staff, the patient's adherence to exercise and to glucose monitoring were assessed by health professionals' interviews.

Quality of Life was assessed by administering patients ad hoc pediatric quality of life questionnaires. More specifically the Euroquol 3D3L young to patients recruited.

Finally, data on Hba1c level, Severe hyperglycemic events rate (number of severe hyperglycemic events averaged over the total number of glycemic readings), Severe hypoglycemic events rate (number of severe hypoglycemic events averaged over the total number of glycemic readings), glycemic variability and glycemic average were collected from CGM data in CGM group or during the hospital visits for SMBG group.

Patient perspective and safety aspects were mainly assessed by patients' interviews. Finally, for cost-effectiveness analysis data were collected from different sources: hospital database, EHR, patients' interview and literature.

Table S2. Data collection methods.

**KPIs**		**Data collection methods**
Organizational aspects		
Workflow	Hospitalization rate	Hospital database
	Length of stay	Hospital database
	Time per consultation	Interview to medical staff
	Number of telephone consultation	Interview to medical staff
	Number of extra visits	Hospital database
Training	Time to download	Interview to medical staff
	Time for data evaluation	Interview to medical staff
	Staff on training	Interview to medical staff
Clinical effectiveness		
Behavioural outcomes	Adherence to exercise	Patients' Interview
	Adherence to glucose monitoring.	Data collected during patients visits from CGM and patients' notes for SMBG
Clinical outcomes	Glycated haemoglobin levels	Data collected during patients visits from CGM and patients' notes for SMBG
	Number of severe hyperglycaemias	Data collected during patients visits from CGM and patients' notes for SMBG

	Number of visits at emergency department	Hospital's database/ EHR
	Number of hypoglycaemic events	Data collected during patients visits from CGM and patients' notes for SMBG
	Number of DKA	Hospital's database/ EHR
	Glycaemic variability	Data collected during patients visits from CGM and patients' notes for SMBG
	Glycaemic average	Data collected during patients visits from CGM and patients' notes for SMBG
Costs and economic evaluation		
	Cost-effectiveness	Hospital's database/ EHR/Patients' interview and Literature
Patient perspectives		
	Adherence to exercise	Patients' Interview
	Adherence to glucose monitoring.	Data collected during patients visits from CGM and patients' notes for SMBG
	Health related quality of life	Patients' Interview
Safety		
Technology related risks	Adverse events	Patients' Interview
